# Left Ventricular Regional Wall Motion Abnormality in the Setting of Acute Loperamide Overdose

**DOI:** 10.5811/cpcem.2019.4.42510

**Published:** 2019-07-01

**Authors:** Kasha Bornstein, Timothy Montrief, Mehruba Anwar Parris

**Affiliations:** * University of Miami Miller School of Medicine, Miami, Florida; † Jackson Memorial Hospital, Department of Emergency Medicine, Miami, Florida

## Abstract

Loperamide is an inexpensive, over-the-counter antidiarrheal agent with emerging reports of overdose due to its opioid properties. Although it is considered by many patients to be safe, cardiotoxicity has been reported, prompting the United States Food and Drug Administration to release a warning regarding the arrhythmogenic potential of loperamide. We present a case of a 32-year-old male presenting in acute loperamide overdose and subsequent cardiac dysrhythmia with focal wall motion abnormalities on echocardiogram. This finding has not been previously reported in the literature and is unique in this clinical presentation. We also highlight the potential mechanisms for loperamide cardiotoxicity and its challenging management.

## INTRODUCTION

Loperamide is an inexpensive, widely used, nonprescription antidiarrheal. Its mechanism of action involves inhibition of intestinal peristalsis through μ-opioid receptor agonism, calcium channel blockade, calmodulin inhibition, and by decreasing paracellular permeability.^1^ Loperamide is sold under the trade name Imodium in the United States (U.S.). When initially introduced in the 1970s, loperamide was listed as a Schedule II drug, due to concerns over its opioid properties. Loperamide was transferred to Schedule V in 1977, and then decontrolled in 1982 following multiple volunteer studies showing low risk of dependence at therapeutic doses secondary to its poor oral systemic bioavailability and limited central nervous system penetration.^2^–^4^ However, reports of loperamide misuse are increasing, more commonly at very high doses of 70–100 milligrams (mg) per day, either to attenuate the effects of opioid withdrawal or for its euphoric effects.^5^

As loperamide misuse has become more widespread in the past decade, descriptions of significant cardiac dysrhythmias in the overdose setting have emerged. These include ventricular tachycardia and torsades de pointes secondary to the drug’s ability to block cardiac sodium and potassium channels at high doses.^6^–^7^ The U.S. faces an increasing population of opioid-addicted patients with escalating opioid overdose mortality. In the setting of efforts to regulate prescription opioid medication misuse through new legislation, many patients are turning to loperamide as an accessible and inexpensive alternative. An added benefit is the lack of social stigma associated with its use.^8^ It is imperative that healthcare providers be made aware of this emerging trend of loperamide use and its under-recognized cardiac toxicity. We describe one case of loperamide overdose presenting with cardiac dysrhythmia and focal wall motion abnormalities discovered on echocardiogram.

## CASE REPORT

**Image 1:**
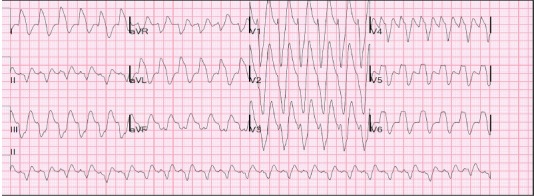
Patient’s initial electrocardiogram showing sinus tachycardia with a left bundle branch block and prolonged QT interval of 328 milliseconds (ms), QTc of 470 ms, and QRS of 140 ms.

A 32-year-old man with a history of polysubstance use including heroin was found combative and delirious in his room by staff at his sober living facility. Multiple empty boxes of loperamide were found in his backpack at the scene by emergency medical services. He was given intravenous (IV) fluids and transported to the emergency department (ED). In the ED, he was notably agitated. On physical exam, he was noted to be tachycardic with a regular pulse of 128 beats per minute and a blood pressure of 123/77 millimeters of mercury (mmHg). He was tachypneic with mild respiratory distress, diaphoretic, and oriented to person only. He was intubated for airway protection using etomidate and succinylcholine. An initial electrocardiogram (ECG) showed a regular, wide-complex tachycardia with a prolonged QTc of 473 milliseconds (ms) and QRS of 140 ms, with an RSR’ pattern in lead aVR. This was determined to be sinus tachycardia with a left bundle branch block by the consulting cardiologist and electrophysiologist (Image 1). The patient’s electrolyte levels were within normal limits.

Acutely, in conjunction with poison control the decision was made to manage the patient with 0.4 milligram (mg) IV naloxone, 400 mg IV lidocaine, and one gram IV magnesium. The patient also received 50 milliequivalent (mEq) IV sodium bicarbonate for presumed sodium channel toxicity from the high-dose loperamide. An initial troponin I was elevated to 0.084 nanograms (ng) per milliliter (mL) (reference 0.000–0.045 ng/mL). A point-of-care ultrasound (POCUS) demonstrated left ventricular anterior wall hypokinesis. He underwent emergent cardiac catheterization, which revealed left ventricular anterior wall hypokinesis, angiographically normal cardiac arteries with a left ventricle ejection fraction (LVEF) of 45%, and an elevated left ventricular filling pressure of 23 mmHg.

**CPC-EM Capsule**

What do we already know about this clinical entity?

Loperamide is a widely used, nonprescription opioid antidiarrheal typically understood to have low risk of dependence given its low bioavailability and limited central nervous system activity.

What makes this presentation of disease reportable?

Prior to this case, there were no reported instances of cardiac angiography confirming loperamide as a primary pharmacological cause of depressed myocardial function.

What is the major learning point?

Loperamide toxicity can present with elevated cardiac injury markers. Resuscitation should be guided by treatment of factors that may induce electrolyte abnormalities.

How might this improve emergency medicine practice?

This case contributes to awareness of the toxidrome as a unique clinical entity. Management to guide resuscitation should be based on pharmacologic principles.

**Image 2:**
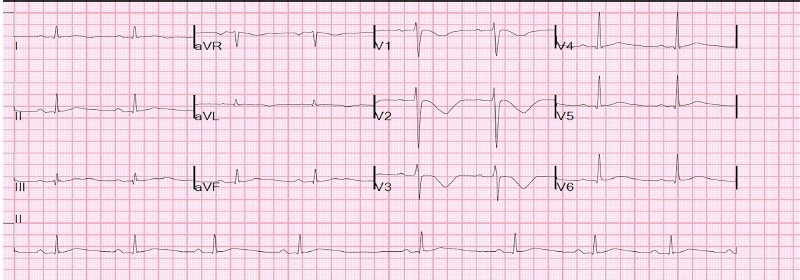
Patient’s electrocardiogram one day after admission showing first degree atrioventricular block, and loperamide-induced QT prolongation, with a QT interval of 616 milliseconds (ms), QTc of 573 ms, and QRS of 120 ms.

**Image 3:**
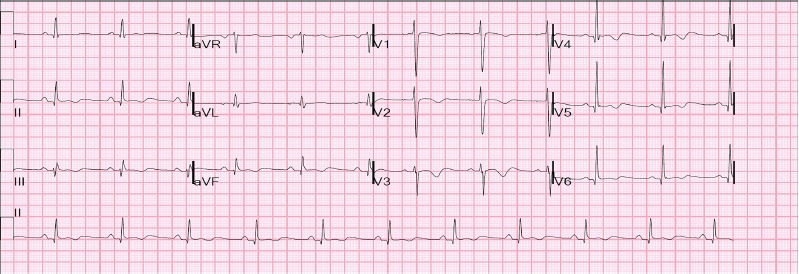
Patient’s electrocardiogram upon discharge, showing a sinus rhythm with a QT interval of 434 milliseconds (ms), QTc of 442 ms, and QRS of 98 ms.

During his hospitalization, the patient’s QT peaked one day after admission to 616 ms with a QTc 573 ms and a QRS of 120 ms (Image 2). At that time the patient admitted to taking over 100 mg of loperamide as well as an unknown amount of gabapentin as an alternative to opioids on the day of admission. His dysrhythmia resolved and QTc normalized within four days of admission with no further exposure to loperamide. A subsequent transthoracic ECG showed a LVEF of 55% and no evidence of diastolic dysfunction or hypokinesis of the anterior wall. At discharge, his ECG showed a QTc of 442 ms and a QRS of 98 ms (Image 3). A loperamide serum concentration was not measured during hospitalization. Urine toxicology screen on admission was negative for opiates as loperamide is not included in the standard panel of drugs screened.

## DISCUSSION

This is a novel case of acute loperamide overdose with subsequent cardiac dysrhythmia with focal wall motion abnormalities on ECG. We do not think gabapentin contributed to his cardiac toxicity as the mechanism of action, nor would toxicity of gabapentin explain the clinical presentation or the laboratory abnormalities. Furthermore, there have been no prior reports of arrhythmias with gabapentin, although both hypertension and hypotension have been reported. The management of loperamide toxicity is largely supportive, although some recommendations can be extrapolated from case reports, pharmacologic principals, and anecdotal experience. In the setting of an acute ingestion, loperamide should adsorb to activated charcoal. Activated charcoal can be administered within two to four hours after a large overdose, provided the patient is not an aspiration risk, in contrast to our case.^9^

For patients with decreased responsiveness or respiratory depression, naloxone can be considered as an adjunct to appropriate airway management. This modality showed benefit in one adult case^10^ and a series of pediatric cases.^11^,^12^ In one pediatric case, a four-month-old girl inadvertently ingested approximately two mg/kg of loperamide liquid and subsequently became comatose with respiratory depression. She recovered after three injections of 0.01 mg IV naloxone over 24 hours.^12^ While naloxone should reverse the respiratory depression induced by loperamide’s μ-opioid agonism, it would not be expected to affect the cardiotoxicity associated with loperamide. However, little data exists to guide naloxone use in acute loperamide toxicity, including dosing. Given loperamide’s long half-life, repeated doses of naloxone may be warranted.^12^

ECG abnormalities are characteristic of loperamide toxicity, particularly prolonged QT and QRS intervals. Again, little data exists to guide cardiac resuscitation in the setting of loperamide ingestion. It is therefore reasonable to treat factors that may induce ECG interval prolongation such as electrolyte abnormalities and other medications. This may be attempted with antiarrhythmic medications such as amiodarone, sotalol, lidocaine, or procainamide,^13^–^15^ as well as sodium bicarbonate to overcome loperamide’s sodium channel blockade. Class 1b antiarrhythmic agents such as lidocaine may be preferable in loperamide toxicity as they have weak sodium channel blocking effects and decrease the effective refractory period in comparison to other class one agents.

## CONCLUSION

Within the literature, the majority of case reports regarding loperamide overdose describe a constellation of symptoms including decreased levels of consciousness, unheralded syncope, markedly increased QTc and QRS intervals, and dysrhythmias including ventricular tachycardia and torsades de pointes. Only one case is reported in which a long-time loperamide user presented with severely depressed global left ventricular function measured by ECG but normal coronary angiography.^16^ Additionally, two case reports of loperamide overdose described mild global hypokinesis of the left ventricle as measured by ECG without corresponding coronary angiography.^15^,^17^ The literature includes only one case report of acute loperamide overdose involving a patient who received both an ECG and coronary angiography. In that case, investigations revealed normal ventricular function and no angiographic abnormalities.^18^ This case provides an opportunity to describe a novel presentation of acute loperamide toxicity, with an elevated cardiac injury marker and confirmed focal left ventricular anterior wall hypokinesis that subsequently resolved after clearance of the drug.
